# The impacts of anxiety and depressive symptoms on emotional processing in children and their parents: an event-related potential study

**DOI:** 10.1186/s13034-023-00610-1

**Published:** 2023-05-11

**Authors:** Zhuo Rachel Han, Julia Yan, Xuan Yang, Mingjia Guo, Kara Braunstein West, Cynthia Suveg, Hui Wang

**Affiliations:** 1 Beijing Key Laboratory of Applied Experimental Psychology, National Demonstration Center for Experimental Psychology Education, Faculty of Psychology, Beijing Normal University, Beijing, China; 2 Department of Human Development and Family Studies, The University of Tennessee, Knoxville, TN USA; 3 Department of Psychology, University of South Carolina, Columbia, SC USA; 4 School of Humanities and Social Sciences, Beijing Institute of Technology, Beijing, China; 5 Children’s Hospital of Philadelphia, Philadelphia, PA USA; 6 Department of Psychology, University of Georgia, Athens, GA USA; 7 School of Applied Psychology, Beijing Normal University at Zhuhai, No. 18 Jinfeng Road, Zhuhai, 519087 China

**Keywords:** Late positive potential (LPP), Emotional processing, Parent-child dyad, Anxiety, Depression

## Abstract

**Background:**

Anxiety and depressive symptoms are associated with dysregulated emotional processing. However, less is known about the intra-personal and inter-personal impacts of anxiety and depressive symptoms on emotional processing in children and their parents.

**Methods:**

In a community sample of 36 parent-child dyads (total *N* = 72), the current study investigated the intra- and inter-personal effects of anxiety and depressive symptoms on the child’s and the parent’s neurophysiological responses to emotional (i.e., pleasant and unpleasant) stimuli, indexed by the late positive potential (LPP).

**Results:**

The results indicated that children’s anxiety symptoms were correlated with their enhanced LPPs to pleasant versus neutral pictures. Additionally, children’s depressive symptoms related to their increased LPPs to unpleasant stimuli. Importantly, children’s anxiety symptoms were associated with their parents’ increased LPPs to both unpleasant and pleasant information.

**Conclusions:**

These findings suggest that anxiety symptoms in community children were related to their own as well as their parents’ emotional processing. The findings contribute to cognitive and family models of anxiety and depression and further highlight the potential role of dyadic interventions for the alleviation of impairing symptoms in children and their caregivers.

## Background

Anxiety and depressive symptoms are among the most common mental health concerns for both children [1, 2] and adults [3]. Heightened levels of anxiety and depressive symptoms, even at the subclinical level, place individuals at risk for concurrent and subsequent difficulties in social competence (e.g., insecure attachment and negative parenting, [4]) and physical well-being (e.g., cardiovascular disease, [5]). Treatment effects are generally modest for internalizing disorders in both children and adults, suggesting the need to further identify potentially malleable targets for intervention [6, 7].

According to the cognitive models of psychopathology [8], one way various forms of psychopathology are manifested is through disrupted processing of emotional information [9, 10]. Event-related potentials (ERPs), recorded from the scalp through electroencephalogram (EEG), provide an excellent way to study neural activity related to emotional processing as well the specific abnormalities underlying psychopathological symptoms given high temporal resolution [11, 12]. In particular, the late positive potential (LPP), a positive component of the ERP, appears approximately 300 ms following the onset of emotional stimuli and tends to reach a maximum at centroparietal recording sites [13, 14]. The LPP is thought to reflect sustained attention to and elaborative processing of emotional stimuli in both children [11, 15] and adults [16].

The LPP has a greater amplitude to both pleasant and unpleasant stimuli than to neutral stimuli in community [11] and clinical samples [17, 18]. Moreover, the magnitude of the difference between emotional versus neutral stimuli is thought to reflect individual differences in emotion processing and has been shown to relate to symptoms of psychopathology, such as anxiety [15] and depression [19]. Studying neural correlates of normal and abnormal emotional processing may help to identify risk processes in the emergence of emotional problems and psychopathology. Prior studies have examined these processes in clinical and healthy, non-disordered samples [19]. However, much information is lost about how these processes operate along the continuum of normal and abnormal development. The present study addresses this critical research gap and examines the relation between symptoms of psychopathology and neural correlates of emotional processing in a community sample of children and their parents who are experiencing a range of symptoms.

### Intrapersonal effects of symptoms and the LPP response

Individuals with symptoms of anxiety and depression show abnormalities in their LPP response. Cognitive models of anxiety, and the hypervigilance hypothesis in particular, posit that anxious individuals may overly attend to threatening information in their environment [20]. Consistent with this view, greater levels of anxiety symptoms have been shown to relate to increased LPPs to unpleasant stimuli [21, 22]. For example, Solomon and colleagues examined the associations between temperamental fear and anxiety and LPP amplitudes in typically developing 5-7-year-old children [15]. Results showed that larger LPP amplitude difference between unpleasant and neutral pictures positively correlated with greater observed fearful behaviors. Similarly, increased LPP amplitudes following the onset of unpleasant versus neutral pictures were found among adults with anxiety disorders [12, 16] and high levels of trait anxiety [23]. Collectively, anxiety is consistently linked with hypervigilance towards threat-related information as evidenced by enhanced LPPs to unpleasant stimuli.

Less work has considered whether anxious individuals present attentional biases when processing pleasant-related information. Evidence from behavioral and neuroimaging studies demonstrates that anxiety-related attentional biases may also be observed in response to pleasant stimuli [24, 25]. According to emotionality hypothesis [26], the hypervigilance pattern in anxiety may not be specific to threats but to emotional information in general; that is, anxiety-related attentional biases may also be observed in response to pleasant materials. Indeed, some studies have reported an anxiety-related bias for pleasant stimuli [27, 28]. For example, Burkhouse et al. found that female undergraduates with high levels of worry displayed increased LPP amplitudes in response to pleasant compared to neutral stimuli when completing a passive viewing task [28]. However, null results have also been reported in studies of anxiety. Specifically, adults with anxiety disorders [29], children with higher levels of parent-reported anxiety symptoms [15, 30], and children with current anxiety disorders [21] exhibited no difference in LPP amplitudes when processing pleasant or neutral stimuli. For instance, McLean et al. [30] found that anxiety problems in 4 years old children were not associated with differences in LPP responses between pleasant and neutral stimuli. Potential reasons for these mixed findings across studies may be the various types of stimuli used for emotional induction and the different methods used to assess emotional processing biases.

In contrast to anxiety, cognitive models of depression suggest that individuals with depressive symptoms may exhibit a decreased attention to positive emotions [31, 32]. Several studies have found that adults with nonclinical depressive symptoms [33, 34] and with major depressive disorder [29, 35] exhibit reduced LPPs to happy and rewarding pictures. Similar to the results of adult studies, there is evidence to suggest that both clinically depressed children [19, 36] and children with higher depressive symptoms [37, 38] show reduced LPPs to pleasant stimuli.

However, the extant literature has conflicting opinions as to whether individuals with depressive symptoms may exhibit excessive attention toward negative emotions [39], or are instead characterized by blunted processing of unpleasant stimuli emotional responses to negative stimuli [32]. For instance, Jaworska et al. found that adults with a major depressive disorder displayed enhanced LPPs in response to sad faces [40]. Nonetheless, depression has also been related to adults’ attenuated LPP responses when processing aversive pictures [12, 33] or anger faces [41]. Similarly, inconsistent results have been found when children process unpleasant stimuli. Specifically, Auerbach et al. found adolescents aged 13–18 with major depressive disorder displayed an enhanced LPP when processing self-referential negative words [42]. By contrast, in a community sample of 3-year-old children, a greater degree of sadness predicted reduced LPP reactivity to unpleasant pictures 6 years later [43]. Inconsistent findings highlight the need to further clarify the relation between depressive symptoms and the LPP in response to unpleasant stimuli. Doing so is crucial for refining models of depression and also identifying potentially malleable targets of intervention.

### Interpersonal effects of symptoms and the LPP response

Both anxiety and depressive symptoms and disorders, which reflect deficits in emotion regulation, are familial. To date research has primarily focused on identifying genetic and environmental correlates and/or contributions [44, 45]. Family systems theory suggests that family members are necessarily interdependent and exert reciprocal impacts on one another [46] and emerging work suggests that family members may also influence one another’s neural responses to emotional stimuli [47–49]. For example, van den Heuvel et al. found that 4-year-old children who were prenatally exposed to higher maternal anxiety displayed greater LPPs to neutral pictures at age 4 [50]. According to cognitive models of anxiety [20], young children at risk for anxiety symptoms may be hypervigilant for threat, and in turn perceive ambiguous stimuli as threatening. In another study, Nelson et al. showed that parents’ anxiety disorders (particularly fear disorders) were associated with 13-15-year-old children’s increased LPPs to unpleasant pictures [48]. These findings are in line with both family systems theory and cognitive models of psychopathology, and suggest that the effects of anxiety symptoms on emotional processing may operate at the interpersonal level.

However, in parallel with the literature on the intrapersonal influence of depression on LPP responses to unpleasant stimuli, findings related to interpersonal effect are also mixed. Several studies have provided evidence that depressive symptoms in parents are correlated with a reduced LPP to both unpleasant and pleasant stimuli in offspring [43, 48, 51]. However, others have shown the opposite pattern, namely a greater LPP to unpleasant stimuli among children with a maternal history of depression [49]. Given the dearth of literature examining relations between parents’ symptoms and children’s neural responses, additional research is needed to understand how parents’ symptoms confer risk for children’s abnormalities in emotional processing at the neural level.

Consistent with family systems theory [46], children may have reciprocal and adverse consequences on their parents’ emotional functioning due to the bidirectional nature of parent-child interactions [52, 53]. However, no study, to our knowledge, has yet examined the influences of child psychopathology on parental LPPs to emotional stimuli. Despite lack of direct evidence, related studies suggest that children’s problematic characteristics, such as frequent and intense distress and depression, are likely to heighten parents’ negative emotionality [54, 55]. Thus, it is reasonable to expect that child psychopathological symptoms might increase the parental risk for abnormalities in emotional processing.

### The present study

The current study investigated the intra- and inter-personal effects of anxiety and depressive symptoms on neural responses to emotional stimuli in a community sample of parent-child dyads. This study makes a substantive contribution by using a rigorous methodological approach to test hypotheses that integrate both cognitive and family theories. Such basic research is needed to best inform a promising approach to improve the utility of cognitive interventions to prevent and relieve psychopathological symptoms [56].

Given extant literature and based on cognitive and family-systems theories, we expected that children and parents would show a higher amplitude LPP to both unpleasant and pleasant pictures compared to neutral pictures. However, we expected the degree of the amplitude to vary as a function of parent and child symptoms. On the intrapersonal level, consistent with the hypervigilance hypothesis [20], we expected that parents and children with higher levels of anxiety symptoms would display enhanced LPP amplitudes to pleasant and unpleasant pictures. On the other hand, based on cognitive models of depression [31, 32] and previous findings [19, 33], we expected that parents and children with higher levels of depressive symptoms would show decreased LPPs to pleasant pictures. On the interpersonal level, consistent with the ideas proposed by the family systems theory [46] and previous studies [50, 51], we expected that parents’ and children’s symptoms would be related to their partner’s LPP responses to emotional stimuli. However, because of the dearth of literature in this area, and conflicting findings among the few existing studies we did not have specific hypotheses about the directions of the effects.

## Methods

### Participants

Thirty-nine parent-child dyads participated in the current study. Participants were recruited through online advertisements and flyers distributed in the community. Children were between 7 and 12 years old (20 boys and 19 girls). Parents were between 33 and 45 years old (30 biological mothers and 9 biological fathers) and self-identified as the primary caregivers. Three dyads were excluded from analysis due to poor quality of recordings. The final sample consisted of 36 children (*M* = 9.01 years, *SD* = 1.85 years; 18 boys and 18 girls), and their parents (*M* = 39.28 years, *SD* = 2.40 years; 28 mothers and 8 fathers). Most families (86.1%) had an annual household income at or above the average family income of the city (i.e., 150,000 RMB, approximately 21,800 USD; [57]). Most parents had a bachelor’s degree or higher level of education (91.7%) and were married (97.2%) at the time of the study. All parent-child dyads were Chinese Han ethnicity.

### Procedures

During the laboratory visit, written informed parental consent and child assent were obtained upon arrival. Parent-child dyads were then asked to complete self-report questionnaires regarding their anxiety and depressive symptoms. Research assistants read questionnaires aloud to children and clarified any questions to ensure understanding. Then, children and parents completed the passive viewing task examining their neurophysiological responses to emotional stimuli for the electroencephalogram (EEG) session. Participants sat in a comfortable chair in a dimly lit and sound-attenuated room, and electrodes were affixed to the scalp of the child and the parent. Participants were instructed to passively view 90 emotional pictures displayed on the screen while EEG signals were recorded. To reduce any effects of dyadic interactions on one’s emotional responses, the parent was not in the room when the child was completing the task, and vice versa. The entire visit lasted 3 h. Families received 500 RMB (approximately 73 USD) for the lab visit.

### Measures

#### Child anxiety symptoms

Children reported on their anxiety symptoms using the Screen for Child Anxiety Related Emotional Disorder [58]. The scale is composed of 41 items rated on a 3-point Likert scale (1 = almost never, 2 = sometimes, 3 = often). The SCARED Total score is calculated by summing all 41 items, and a higher total score indicates higher child anxiety symptoms. The original SCARED has satisfactory psychometric properties [58], and the Chinese version also shows test-retest reliability and good internal consistency [59]. Furthermore, it has been reported to be robust in both clinical and community samples [60, 61]. For current study, the SCARED showed good reliability (*α* = 0.88).

#### Child depressive symptoms

Child depressive symptoms were assessed with the 20-item Center for Epidemiological Studies in Depression Scale (CES-D; [62]). Children reported on their depressive symptoms over the previous week on a 4-point Likert scale: 1 = rarely or none of the time (less than 1 day), 2 = some or a little of the time (1–2 days), 3 = occasionally or a moderate amount of time (3–4 days), and 4 = most or all of the time (5–7 days). Total scores range from 20 to 80, with higher scores indicating higher levels of depressive symptoms. The original CES-D is well established [62] and has been utilized as a reliable and valid measure of Chinese children’s depressive symptoms [63]. The internal consistency of the CES-D in the current study was *α* = 0.82.

#### Parental anxiety and depressive symptoms

Parents completed the Symptom Checklist-90-Revised (SCL-90-R; [64]) to report on psychopathological symptoms experienced over the previous week. Parents respond on a 5-point Likert scale ranging from 1 (not at all) to 5 (extremely). For the purposes of the current study, the 10-item Anxiety subscale and the 13-item Depression subscale were used. Items were summed and *T* scores were computed, with higher scores indicating higher distress. The SCL-90-R has well-established reliability and validity [64] and has been validated with Chines samples [65]. In the current study, the internal consistencies for the anxiety and depression subscales were *α* = 0.92 and *α* = 0.89, respectively.

### Passive viewing task

The passive viewing task occurred after EEG set up. A total of 90 developmentally appropriate pictures were selected from the International Affective Picture System (IAPS; [66]). Of these, 30 depicted unpleasant scenes (e.g., airplane crashes, threatening animals), 30 depicted pleasant scenes (e.g., cute animals and babies), and 30 depicted neutral scenes (e.g., natural scenery, household objects)^1^. Stimuli were presented using the EEGLAB software toolbox for MATLAB. Thirty pictures were randomly selected for each experimental block over a total of 3 blocks. Each picture was randomly presented once and occupied the entire 14.1” screen. Each trial began with an instruction (“Simply view these pictures”) for 2000 ms, then each picture was presented for 4500 ms followed by a fixation point (“+”) for 500 ms.

### EEG recording and data reduction

The continuous electroencephalogram (EEG) was recorded throughout the passive viewing task using a Neuroscan Synamp2 Amplifier. Recordings were taken from 64 cap-mounted Ag/AgCl electrodes (10/20 system). The electrooculogram (EOG) generated from eye blinks and movements was recorded from four electrodes: two electrodes attached to the outer canthus of each eye to monitor the horizontal EOG and two electrodes placed approximately 1 cm above and below the left eye to monitor the vertical EOG. The EEG was sampled at 500 Hz. The impedance of all electrodes was maintained below 5 kΩ. All EEG signals were referenced to the left mastoid and were bandpass filtered at 0.05–100 Hz during data collection.

Offline analysis was performed using Neuroscan4.3 software. All data were rereferenced to the average of the left and right mastoids and bandpass filtered with cutoffs at 0.1 and 30 Hz. Eyeblinks were corrected offline using a regression procedure [67]. Data were segmented for each trial, beginning 300 ms before and continuing 3500 ms after each picture onset. ERPs were baseline corrected using the 300 ms prior to the stimulus. The semiautomated artifact removal procedure excluded any segment with voltage steps exceeding ± 100 µV from further analyses. Additional artifacts were detected using visual inspection. Three dyads were excluded from the analyses because of excessive artifacts (averaged rejected epochs more than 50%).

Based on a visual inspection and previous work [14, 34, 68], the LPP was computed as the mean amplitude of the EEG in a 500–1000 ms time window. The LPP was then averaged in three regions: posterior (Pz, P3, P4, Oz, O1, O2), central (Cz, C3, C4, CPz, CP3, CP4), and anterior (Fz, F3, F4, FCz, FC3, FC4).

### Data analyses

First, repeated-measures analyses of variance (ANOVA) were conducted in SPSS 21.0 to evaluate the LPP across each picture type (i.e., unpleasant, pleasant, and neutral pictures). Greenhous-Geisser corrections were applied when assumptions of sphericity were violated. Post hoc multiple comparisons were conducted using the Bonferroni correction. Effect sizes were measured as partial eta-squared (${\eta }_{p}^{2}$). Second, the descriptive statistics and correlations among parent-child LPP amplitudes and anxiety and depressive symptoms, and possible group differences based on demographic characteristics were reported using SPSS 21.0.

**Fig. 1:**
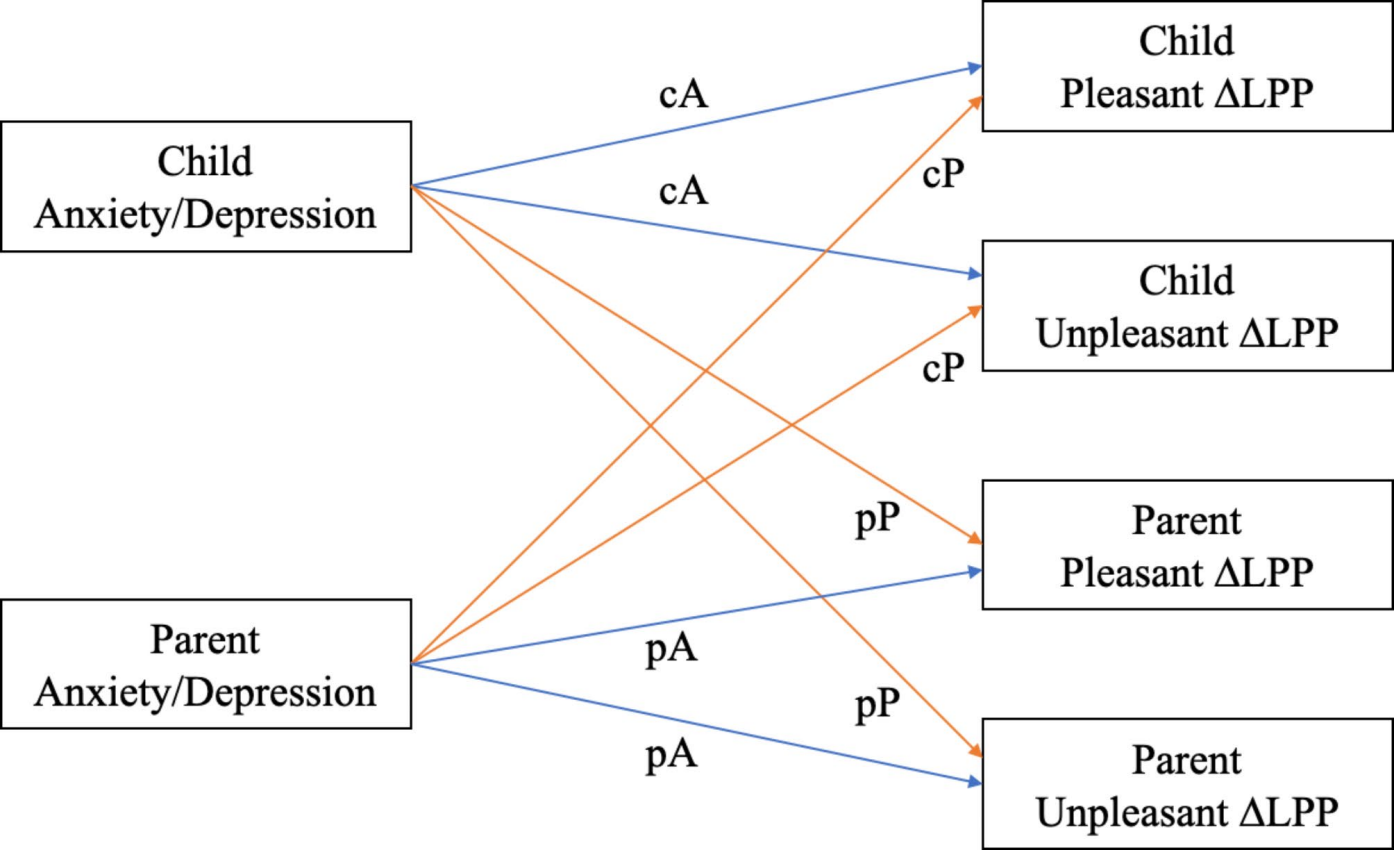
The APIM model of the intra- and inter-personal impacts of anxiety and depression on parents’ and children’s LPP responses to pleasant and unpleasant pictures compared to neutral pictures *Note*. The intra-personal effects included the child actor effect (cA) and the parent actor effect (pA), while the inter-personal effects included the child partner effect (cP) and the parent partner effect (pP)

Finally, the Actor-Partner Interdependence Models (APIM; [69]) was employed in Mplus 7.0 to investigate the effects of parental and child anxiety and depressive symptoms on their own (i.e., intrapersonal) and their partner’s (i.e., interpersonal) LPP responses to unpleasant and pleasant pictures compared to neutral pictures. The APIM is well-suited for analyzing dyadic data as it accounts for the non-independence of the data within the actor-partner interdependence model [69]. We aimed to investigate the separate and independent impacts of anxiety and depression on children’s and their parents’ neural responses to emotional stimuli, and thus, two APIMs were utilized: one model predicted LPP responses to pleasant and unpleasant stimuli relative to neutral stimuli based on children’s and parents’ anxiety levels, while the other model predicted LPP amplitudes with depression levels as the predictor variable (see Fig. 1). Given the number of comparisons (total analyses = 2), the Bonferroni correction was implemented to decrease the likelihood of Type I errors (*α =* 0.025). Missing data were handled with the full-information maximum likelihood.

## Results

### LPP amplitudes of children and parents

**Fig. 2:**
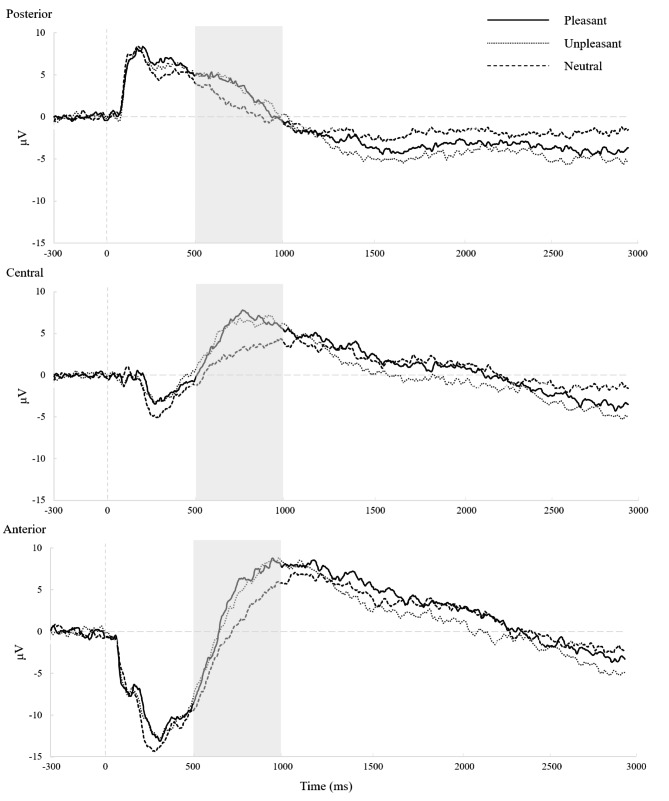
Child LPP waveforms for unpleasant, pleasant, and neutral pictures at posterior, central, and anterior regions

Two 3 (picture type) × 3 (region) repeated-measure ANOVAs were performed separately for parents and children. With respect to children, significant main effects were found for picture type, *F* (2, 50) = 11.25, *p* < .001, ${ \eta }_{p}^{2}$= 0.31 (see Fig. 2). Unpleasant and pleasant pictures elicited larger LPPs than neutral pictures (mean difference = 2.58, 95% CI = [0.78, 4.37], *p* = .003 for unpleasant pictures; mean difference = 2.69; 95% CI = [1.06, 4.32], *p* < .001 for pleasant pictures). Main effects were also found for region (*F* (2, 50) = 8.79, *p* = .003, ${ \eta }_{p}^{2}$= 0.26); the LPP in the central region was significantly larger than that in the anterior region (mean difference = 3.20; 95% CI = [1.91, 4.48], *p* < .001). There was no interaction between picture type and region (*F* (4, 100) = 0.72, *p* = .58, ${ \eta }_{p}^{2}$ = 0.03).

**Fig. 3:**
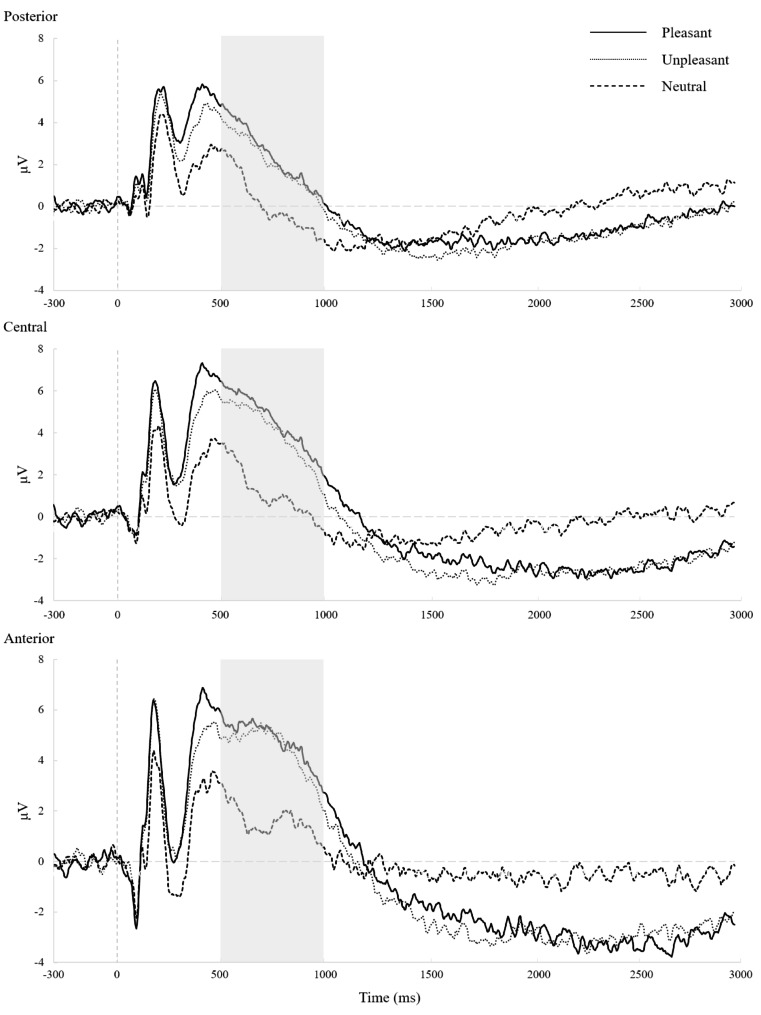
Parental LPP waveforms for unpleasant, pleasant, and neutral pictures at posterior, central, and anterior regions

For parents, the results revealed main effects of picture type (*F* (2, 62) = 33.68, *p* < .001, ${ \eta }_{p}^{2}$= 0.52) and region (*F* (2, 62) = 31.43, *p* < .001, ${ \eta }_{p}^{2}$= 0.50) which were qualified by an interaction between picture type and region (*F* (4, 124) = 4.85, *p* < .001, ${ \eta }_{p}^{2}$ = 0.14; see Fig. 3). Bonferroni pairwise post hoc comparisons indicated that LPP amplitudes in response to both unpleasant and pleasant pictures were significantly larger than those to neutral pictures (mean difference = 2.68; 95% CI = [1.47, 3.88], *p* < .001 for unpleasant pictures; mean difference = 2.98; 95% CI = [2.18, 3.79], *p* < .001 for pleasant pictures). The LPP amplitudes in the anterior and central regions were significantly larger than those in the posterior region (mean difference = 1.94; 95% CI = [1.11, 2.77], *p* < .001 for the anterior region; mean difference = 1.70; 95% CI = [1.10, 2.30], *p* < .001 for the central region). Furthermore, post hoc tests demonstrated that LPP amplitudes elicited by both unpleasant and pleasant pictures were significantly larger than those elicited by neutral pictures in the anterior, central, and posterior regions (*p* < .001 for all comparisons).

Taken together, the results above indicated that for children and parents, unpleasant and pleasant pictures generated larger LPP amplitudes compared to neutral pictures. The LPP appeared to be the largest in the central region for the children and in the central and anterior regions for the parents. Furthermore, it has been reported that LPP amplitudes appear to be more evident at centroparietal sites following emotional versus neutral stimuli [13, 23, 34]. Given these reasons, we focused on the LPP amplitudes at the central regions to test the effects of parental and child psychopathological symptoms. Difference scores were calculated by the relative responses to unpleasant and pleasant compared to neutral pictures (i.e., ∆LPP), with larger LPP difference scores indicating greater LPP amplitudes to emotional stimuli.

### Parent-child anxiety and depression and the LPP

Descriptive statistics and bivariate correlations between study variables were showed in Table 1. Results demonstrated that the mean score for child anxiety symptoms was 58.06 (*SD* = 10.18, range = 42–79), while the mean score for child depressive symptoms was 30.97 (*SD* = 7.86, range = 20 − 3). In addition, the mean score for parent anxiety symptoms was 49.17 (*SD* = 10.66, range = 41–90), and the mean score for parent depressive symptoms was 52.06 (*SD* = 10.42, range = 39–92). The results of the bivariate correlation analysis indicated that child depressive symptoms were positively related to their enhanced unpleasant (*r* = .58, *p* = .003) and pleasant ∆LPP (*r* = .49, *p* = .018). Child anxiety symptoms were marginally correlated with their enhanced unpleasant (*r* = .37, *p* = .071) and pleasant ∆LPP (*r* = .36, *p* = .077), and their parents’ enhanced unpleasant (*r* = .33, *p* = .074) and pleasant ∆LPP (*r* = .34, *p* = .070).

**Table 1:** Descriptive statistics and bivariate correlations among study variables

	*M*	*SD*	1	2	3	4	5	6	7	8	9
1. C-Age	9.01	1.85									
2. C-Gender	0.50	0.51	− 0.14								
3. C-unpleasant ∆LPP	3.41	3.52	− 0.23	− 0.40*							
4. P-unpleasant ∆LPP	2.92	2.81	0.15	− 0.05	0.13						
5. C-pleasant ∆LPP	1.75	6.40	− 0.48*	− 0.11	0.84**	0.03					
6. P-pleasant ∆LPP	3.44	2.16	0.12	0.19	− 0.11	0.61**	− 0.07				
7. C-Anxiety	58.06	10.18	− 0.18	0.03	0.37^†^	0.33^†^	0.36^†^	0.34^†^			
8. P-Anxiety	49.17	10.66	− 0.07	0.24	0.29	− 0.09	0.12	− 0.02	0.26		
9. C-Depression	30.97	7.86	− 0.19	− 0.05	0.58*	0.07	0.49*	− 0.25	0.40*	0.41*	
10. P-Depression	52.06	10.42	− 0.14	0.35*	0.08	− 0.18	0.07	0.05	0.07	0.84**	0.14

***Note.*** C, Children; P, Parents; Unpleasant ∆LPP, the LPP amplitudes to unpleasant versus neutral pictures; Pleasant ∆LPP, the LPP amplitudes to pleasant versus neutral pictures

a Gender was coded as 0 for boys and 1 for girls

^†^*p* < .10, * *p* < .05, ** *p* < .01

We also tested the influences of demographic factors on study variables. Child age was negatively associated with their pleasant ∆LPP (*r* = − .48, *p* = .013). Independent sample t-tests demonstrated that there were child gender differences on their unpleasant ∆LPP (*t* (34) = 2.08, *p* = .046) and on their parents’ depressive symptoms (*t* (32) = -2.11, *p* = .043). Specifically, for boys, unpleasant pictures produced larger LPP amplitudes compared to neutral pictures. Boys’ parents self-reported lower depressive symptoms. However, parental age and gender was not significantly correlated with any study variables. Thus, children’s age and gender were included in the regression as covariates.

### Intrapersonal and interpersonal impacts of anxiety symptoms

For the model with parental and child anxiety symptoms predicting both pleasant and unpleasant ∆LPP, the model fit the data, *χ*^2^ (4) = 3.41, *p* = .49, *CFI* = 1.000, *TLI* = 1.043, *RMSEA* = 0.001. Results (see Table 2) indicated that children’s anxiety symptoms were positively associated with their pleasant ∆LPP (*β* = 0.441, *SE* = 0.170, *p* = .010). Moreover, children anxiety symptoms were positively correlated with their parents’ pleasant ∆LPP (*β* = 0.440, *SE* = 0.162, *p* = .007) and their parents’ unpleasant ∆LPP (*β* = 0.440, *SE* = 0.145, *p* = .002). These significant associations remained significant even after accounting for multiple testing using the Bonferroni correction.

**Table 2:** APIM Analyses with Parental and Child Anxiety Symptoms Predicting ΔLPP

Effect	*β*	*SE*	*95% CI*
Intrapersonal Effect			
Child Anxiety → Child pleasant ΔLPP	0.441*	0.170	[0.059, 0.735]
Child Anxiety → Child unpleasant ΔLPP	0.363	0.207	[-0.096, 0.731]
Parent Anxiety → Parent pleasant ΔLPP	− 0.254	0.250	[-0.672, 0.309]
Parent Anxiety → Parent unpleasant ΔLPP	− 0.329	0.203	[-0.712, 0.080]
Interpersonal Effect			
Child Anxiety → Parent pleasant ΔLPP	0.440**	0.162	[0.077, 0.709]
Child Anxiety → Parent unpleasant ΔLPP	0.440**	0.145	[0.133, 0.701]
Parent Anxiety → Child pleasant ΔLPP	− 0.051	0.224	[-0.475, 0.426]
Parent Anxiety → Child unpleasant ΔLPP	0.174	0.210	[-0.240, 0.605]

***Note.*** Unpleasant ∆LPP, the LPP amplitudes to unpleasant versus neutral pictures; Pleasant ∆LPP, the LPP amplitudes to pleasant versus neutral pictures. Child age and gender are controlled for the parent and the child pleasant and unpleasant ΔLPP. The standardized coefficients are reported. CI, confidence interval

* *p* < .05, ** *p* < .01

### Intrapersonal and interpersonal impacts of depressive symptoms

For the model with parental and child depressive symptoms predicting both pleasant and unpleasant ∆LPP, the model fit the data, *χ*^2^ (4) = 5.81, *p* = .21, *CFI* = 0.974, *TLI* = 0.857, *RMSEA* = 0.075. As shown in Table 3, children’s depressive symptoms were related to their increased unpleasant ∆LPP (*β* = 0.53, *SE* = 0.07, *p* = .001). Moreover, this significant association survived the Bonferroni correction for multiple testing. However, parental and child depressive symptoms did not relate to their own and their partners’ LPP amplitudes to pleasant compared to neutral pictures (see Table 3).

**Table 3:** APIM Analyses with Parental and Child Depressive Symptoms Predicting ΔLPP

Effect	*β*	*SE*	*95% CI*
Intrapersonal Effect			
Child Depression → Child pleasant ΔLPP	0.378	0.184	[-0.029, 0.693]
Child Depression → Child unpleasant ΔLPP	0.505***	0.150	[0.121, 0.725]
Parent Depression → Parent pleasant ΔLPP	0.081	0.226	[-0.375, 0.542]
Parent Depression → Parent unpleasant ΔLPP	− 0.180	0.216	[-0.359, 0.438]
Interpersonal Effect			
Child Depression → Parent pleasant ΔLPP	− 0.208	0.181	[-0.576, 0.133]
Child Depression → Parent unpleasant ΔLPP	0.077	0.205	[-0.359, 0.438]
Parent Depression → Child pleasant ΔLPP	− 0.104	0.231	[-0.598, 0.341]
Parent Depression → Child unpleasant ΔLPP	0.001	0.241	[-0.563, 0.367]

***Note.*** Unpleasant ∆LPP, the LPP amplitudes to unpleasant versus neutral pictures; Pleasant ∆LPP, the LPP amplitudes to pleasant versus neutral pictures. Child age and gender are controlled for the parent and the child pleasant and unpleasant ΔLPP. The standardized coefficients are reported. CI, confidence interval

*** *p* < .001

## Discussion

This study expanded the existing literature by examining the intra- and inter-personal impacts of anxiety and depressive symptoms on neural responses to emotional stimuli within parent-child dyads. This work is needed to refine existing cognitive and family models of anxiety and depression and identify potentially malleable targets for intervention. The results revealed that, on the intrapersonal level, community children’s (but not parents’) anxiety symptoms were associated with their increased LPPs to pleasant stimuli. Children’s depressive symptoms related to their enhanced processing of unpleasant stimuli. Importantly, on the interpersonal level, we found important roles of children’s anxiety symptoms on their parents’ neural modulations of emotional information within families. Specifically, children’s anxiety symptoms were associated with their parents’ increased LPPs to both unpleasant and pleasant pictures compared to neutral ones.

The first goal of the present study was to investigate the neural correlates of anxiety and depressive symptoms and one’s own emotional processing in a sample of community parent-child dyads. Our hypotheses were partially confirmed, showing that children with higher levels of anxiety symptoms displayed more detailed processing of pleasant information, as indicated by greater LPP amplitudes to pleasant compared to neutral pictures. This finding supports the emotionality hypothesis (Mogg & Marden, 1990), demonstrating that anxious children exhibit heightened neural reactivity to emotional stimuli in general, rather than a specific thereat-bias. This expands upon existing knowledge concerning the association between anxiety and neural responses to emotional stimuli. Mush of the previous research on anxiety has focused on neural responses to negative emotions and has established a threat-related attentional bias [15, 16, 21, 22]. However, there is evidence, although limited, suggests that some types of anxiety disorder, such as generalized anxiety disorder [70, 71] and high trait anxiety [72], may elicit one’s hypervigilance bias towards happy faces as well. However, no relation was found between children’s anxiety symptoms and their LPP responses to unpleasant pictures, raising the possibility that anxious children’s attentional biases to unpleasant stimuli can be most clearly understood by matching specific anxieties with relevant stimuli [73].

In addition, we found that children with higher depressive symptoms displayed more sustained attention to the unpleasant information, as indicated by greater LPP amplitudes to unpleasant compared to neutral pictures. Literature has been mixed regarding depression and emotional reactivity to unpleasant stimuli, with some studies supporting enhanced LPPs [40, 42], and others supporting attenuated LPPs to unpleasant information [41, 43]. The current study adds to the literature linking depressive symptoms to increased LPPs to unpleasant stimuli. It may be the differences in experimental paradigms among studies (e.g., emotion interrupt task, self-referential task, and emotional oddball task) that make it difficult to generalize their findings. Future studies are needed to verify the influences of different experimental paradigms on the correlates of depressive symptoms and neurophysiological responses to emotional stimuli.

However, no significant relationship was found between parent-child depressive symptoms and the LPP responses to pleasant pictures. This result was unexpected given most of previous studies have reported decreased attention to pleasant information in depression [19, 33, 34]. This inconsistency may relate to methodological differences among studies, such as whether pleasant and unpleasant stimuli are intermingled in the same blocks or presented in separate blocks [72]. The current study, aligned with previous work [11, 15, 30], intermingled unpleasant pictures with pleasant pictures in the same blocks, which might reduce the emotional modulation effects for pleasant information. In this setting, the negative emotions elicited by unpleasant pictures may undermine or override the positive emotional experiences from pleasant pictures. Indeed, our study, as well as that conducted by McLean et al. [30], employed identical experimental design, and both investigations revealed no significant correlation between depression and LPP responses to either pleasant or unpleasant stimuli.

Guided by family systems theory [46], the main goal of the current study was to explore whether one’s anxiety and depressive symptoms were related to their parents’ or children’s emotional modulation of LPPs to emotional stimuli in parent-child dyads. Results demonstrated support for the interpersonal effects of child anxiety symptoms on parents’ LPPs, indicating that children’s anxiety symptoms were associated with elevated LPP responses to pleasant and unpleasant pictures in their parents. This finding was consistent with the hypotheses guided by family systems theory [46]. Particularly, children are not passive respondents to parenting, parental characteristics, and family functioning. Children can play an active role in shaping parental functioning and well-being [74]. It may be that a child’s anxiety symptoms increase his or her parent’s conscientiousness and concern. In turn, parents tend to be vigilant to their children’s emotions and view the child’s emotions as an opportunity for emotional coaching [75, 76]. Another possibility is that sustained attention towards pleasant stimuli is an emotion regulation strategy of parents of children with higher levels of anxiety [70]. Parents who have been exposed to higher levels of child anxiety may consciously allocate their attention to positive cues in the environment to mitigate unpleasant emotional experiences elicited by their children. This finding extends the current literature on attentional bias by showing that within a family that is characterized by a relatively low-risk context (i.e., community-based families), parents of children with more anxiety symptoms exhibit hypervigilance towards emotional stimuli.

Of note, previous research on interpersonal effects has primarily focused on emotional processing in the offspring of anxious parents [48, 50]. For instance, Nelson et al. found that 13-15-year-old children exposed to parental anxiety, especially fear disorders, exhibited heightened LPP responses to unpleasant stimuli [48], which provided support for the hypervigilance hypothesis of anxiety [20, 26]. The current study extends the hypervigilance model of anxiety by suggesting that, similar to children of parents with anxiety, parents of children with greater levels of anxiety symptoms might contribute to their approach motivation and global engagement to emotional information as assessed by the LPP.

Contrary to predictions, parental anxiety and depressive symptoms had no direct influence on their own or their children’s LPPs to emotional pictures. This finding was inconsistent with some previous studies [33, 51, 77]. One potential explanation for this discrepancy is that many of these previous studies have been limited to clinical adult samples [77], whereas we focus on a community adult sample. Possibly, in our community sample, parents with subclinical levels of psychopathology fail to potentiate their own or their children’s dysregulated neural responses to emotional stimuli than their counterparts presenting with clinical levels of disorders [48]. In addition, this discrepancy may also be due to the age-related attenuation in emotional processing. It is assumed that due to the greater efficiency and regulatory control that come with brain maturation, typically developing individuals may exhibit an age-related attenuation of the LPP amplitudes to emotional information [78, 79]. Thus, parents in our community-based sample may have already developed adequate levels of effortful control to reduce the processing of emotional stimuli, leading to no significant relation between symptoms and LPP amplitudes to emotional stimuli. Regardless, this study represents an important step towards identifying risk processes that underlie psychopathology symptoms. More work is needed to either support or refute the current findings and to identify when in the symptom severity continuum such deficits are evidenced.

### Limitations

There are several limitations that should be considered when interpreting our findings. First, although the sample size was large enough to detect LPP responses to emotional stimuli, it was relatively small for the APIM analyses that we used. Studies with more participants are warranted to replicate our preliminary findings. Additionally, in the current study, anxiety and depressive symptoms were highly correlated with each other. As anxiety and depression might frequently co-occur and share etiological causes [80], it was difficult to disentangle their independent influences on emotional processing. Future research is encouraged to explore the potential differences between pure and comorbid psychopathology groups.

### Implications

Despite the limitations above, this study contributes to cognitive and family models of anxiety and depression and further highlight the importance of implementing interventions targeted to alleviate psychopathological symptoms, especially for children with subclinical levels of psychopathology. The results further suggest that novel approaches to intervention consider the dyad-level aimed at facilitating healthy emotional processing in children and their caregivers.

## Conclusions

This study investigated the reciprocal impacts of anxiety and depressive symptoms on the LPP within community-based parent-child dyads. Results indicated that children’s anxiety symptoms correlated with their enhanced LPPs to pleasant compared to neutral stimuli. In addition, children’s depressive symptoms were associated with their decreased LPP responses to unpleasant relative to neutral information. Furthermore, consistent with the hypotheses of family systems theory [46], parents’ modulations of their LPPs were influenced by their children’s anxiety symptoms, that is child anxiety symptoms related to parental enhanced LPPs to both unpleasant and pleasant stimuli.

## Data Availability

The datasets used and/or analyzed during the current study are available from the corresponding author on reasonable request. The contact information of the corresponding author is Hui Wang, huiwang@bnu.edu.cn.

## List of Abbreviations

LPP: Late positive potential

## Footnotes

1 The numbers of IAPS numbers used were: unpleasant (1050, 1120, 1201, 1300, 1321, 1930, 2120, 2130, 2688, 2780, 2810, 2900, 3022, 3230, 3280, 5970, 6190, 6300, 6370, 7380, 9050, 9250, 9421, 9470, 9480, 9490, 9582, 9594, 9600, 9611); neutral (5220, 5711, 5740, 5750, 5800, 5820, 7000, 7002, 7004, 7006, 7009, 7010, 7025, 7031, 7035, 7041, 7050, 7080, 7090, 7100, 7140, 7150, 7175, 7190, 7224, 7233, 7235, 7236, 7595, 7950); pleasant (1460, 1463, 1601, 1610, 1710, 1750, 1811, 1920, 1999, 2070, 2091, 2165, 2224, 2311, 2340, 2345, 2791, 4603, 5831, 7325, 7330, 7400, 7502, 8031, 8330, 8380, 8461, 8490, 8496, 8620).

## Declarations

### Data transparency statement

The work described is original research that has not been published previously and is not under consideration for publication elsewhere, in whole or in part.
